# A comprehensive UK crop yield dataset incorporating satellite, weather, and soil type information

**DOI:** 10.1038/s41597-025-06528-x

**Published:** 2026-02-20

**Authors:** Evangeline Corcoran, Daniel P. Bebber, Stelian Curceac, Natalia Efremova, Azam Lashkari, Andrew Mead, Richard J. Morris, Richard F. Pywell, John W. Redhead, Sebastian E. Ahnert

**Affiliations:** 1 The Alan Turing Institute, 96 Euston Road, London, NW1 2DB UK; 2 Department of Biosciences, University of Exeter, Geoffrey Pope Building, Exeter, EX4 4QD UK; 3 Statistics and Data Science Section, Intelligent Data Ecosystems, Rothamsted Research, Harpenden, Hertfordshire, AL5 2JQ UK; 4 Regional Climate & Hydrology, Karlsruhe Institute of Technology, Kaiser Street 12, 76131 Karlsruhe, Germany; 5 School of Business and Management, University of Queen Mary of London, Mile End Road, London, E1 4NS UK; 6 Department of Computational and Systems Biology, John Innes Centre, Norwich Research Park Colney Lane, Norwich, NR4 7UH UK; 7 UK Centre for Ecology and Hydrology, Maclean Building, Benson Lane, Crowmarsh Gifford Wallingford, OX10 8BB Oxfordshire, UK; 8 Department of Chemical Engineering and Biotechnology, University of Cambridge, Philippa Fawcett Dr, Cambridge, CB3 0AS UK

**Keywords:** Agroecology, Plant ecology, Agroecology

## Abstract

Agricultural research increasingly relies on data-driven approaches for crop yield prediction that complement more established crop growth models, including machine learning techniques. However, these approaches rely on large training datasets. Here, we present the Crop Yields, Climate, Soils, and Satellites (CYCleSS) dataset, a large-scale crop yield dataset derived from precision yield data for 934 fields across England on which a variety of crops are grown. In addition, the data also contains satellite-derived remote sensing data, weather data, and data on soil type, all aligned at a grid resolution of 10 km. Weather data is available at a daily temporal resolution, satellite data at 5-day resolution, while crop yield data is available at yearly resolution. This effort has been made possible through careful anonymisation of the yield data while preserving the alignment with remote sensing, weather, and soil data. This data will be useful both to train machine learning models of yield prediction as well as to parameterize mechanistic crop growth models. Furthermore, the anonymisation procedure itself will be of interest to the research community, as it represents a solution to a common problem on the interface of agricultural research and farming practice.

## Background & Summary

Crop yield prediction is an essential tool for food security research, agricultural resource management, climate risk assessment, climate adaptation, and policy decisions in a changing climate. However, projecting crop yield is fraught with uncertainty, depending on the quality of input data and models at every turn^1^. Crop yield is a complex trait and accurate crop yield prediction requires the use of data on a multitude of variables that have been demonstrated to impact crop plant growth such as climate, soil, and agricultural inputs^2^. The impact of all these variables differs between crop cultivars and genotypes, and changes throughout development. The two major modelling approaches for crop yield prediction are process-based (dynamic) crop models and data-driven approaches^3^. The difference between these two approaches is that process-based models are driven by the physiological principles of plant growth, drawing from the domain expertise and theory of fields such as crop physiology, crop ecology, meteorology, and soil science, while data-driven model parameters are derived directly from the data, using domain knowledge to inform selection of model inputs^4,5^. The importance of collecting data for dynamic crop models has been extensively discussed in a previous paper by Corcoran *et al*.^6^. In this paper we focus on the importance of data for data-driven approaches, with an emphasis on those that apply machine learning.

The term “data-driven modelling” (DDM) refers to the overarching paradigm of using historical data derived from real-world systems in conjunction with advanced computational techniques, including machine learning and artificial intelligence, to create models that can reveal underlying trends and patterns, and potentially predict future outcomes^3,5^. Unlike process-based models, data-driven models can optionally be developed and trained to make predictions without detailed knowledge of the underlying processes governing the system behaviour, which makes these models particularly valuable when knowledge of these processes is incomplete^7,8^. In this regard, machine learning-based approaches have been used with increasing frequency in agriculture and crop yield prediction over the past several years due to their ability to model non-linear processes and complex relationships between multiple inputs^9^. Another advantage of machine learning approaches is that regularisation techniques can be applied to these models, which have been shown to improve accuracy when dealing with noisy data^8^. However, a major trade-off for the flexibility of these approaches is that machine learning algorithms require larger amounts of data compared to process-based models to distinguish between genuine patterns and noise, accompanied by a risk of overfitting^10^.

To reduce the risk of overfitting and improve the generalisability of machine learning approaches, large amounts of high-quality training data are required to train models^11^. Ground truth data, direct observations or measurements that are known to be real and true, are of particular importance when developing machine learning models as they can be used to quantify how accurately models perform, a process commonly referred to as model validation^12^. Ground truth data that can be used to develop and validate machine learning models for crop yield prediction are also potentially beneficial for testing and verifying the accuracy of predictions made with conventional process-based models, as the actual yield observed can be compared to the yield predictions, providing evidence for the robustness and generalisability of process-based models^13^. However, for many scientific domains, scarcity of data on which to train models, and in particular scarcity of ground truth data for use in validating models, is a major constraint on development of machine learning models^14^. A rise in the availability and accessibility of spatiotemporal data with high coverage collected by remote sensors on the climate, soil, and land use variables that impact crop yield has provided a potential way to gather large and varied training datasets for crop yield prediction, but efforts to develop these models thus far have been limited by a lack of ground truth data^5^. Direct observations of yield have often only been available for a small number for fields for limited crops, resulting in a lack of generalisability of machine learning models to areas and crops outside those included in the restricted validation dataset^8^.

The widespread adoption of precision agriculture in recent years has meant that huge volumes of data on crop yield at high spatial resolutions are routinely collected by farm machinery^15–17^. These data are of great potential benefit for training and validating yield models, including machine learning approaches, which can then be used to extrapolate predicted yield beyond the locations and time frames over which precision yield data were collected. Many studies have successfully demonstrated these approaches, using a wide variety of satellite data sources and indices as predictors of yield^18,19^. However, fundamental constraints on building and using such models are the restrictions around access and use of precision yield data. Although large data volumes are widely collected, these data remain the property of individual farmers, so such data are commercially sensitive and not widely accessible. Even when precision yield data are made available to researchers, they are not generally approved for wider publication^19^. This is an issue in common with much data on agricultural practice, from pesticide use^20^ to agri-environmental uptake^21^. Therefore, to use precision yield data effectively in the production of yield models for widespread deployment requires procedures to ensure that the source and location of precision yield data can be effectively anonymised. In this study, we aim to address the scarcity of ground truth data for use in the development of machine learning models and validation of process-based models for yield prediction for crops in the United Kingdom (UK). To address this issue, we have assembled a dataset of anonymised yield data derived from precision agriculture, as well as data on crop type, soil conditions, daily time-series of climate data and 5-day time-series of Sentinel-1 Radar Band data for 2015–2017. This Crop Yields, Climate, Soils, and Satellites (CYCleSS) dataset will provide invaluable resources for crop yield prediction across the UK and its relative issues. It will also help to promote the FAIR Guiding Principles (in which data should be findable, accessible, interoperable, and reusable)^22^ and in facilitating data-driven modelling in environmental and agricultural research.

A potential use of the CYCLeSS dataset is in the development of machine-learning based models to predict field-scale crop yield for the UK. The CYCLeSS dataset may be particularly beneficial for the development of deep learning approaches, such as convolutional neural networks (CNN), deep neural networks (DNN), and long-short term memory models (LSTM). These methods have been found to produce accurate and efficient results when previously used to predict crop yield in other geographic areas and for different scales^23–25^. There are two main aspects of the CYCLeSS that address current data gaps that have hindered development of a large-scale crop yield model for the UK. Firstly, a lack of ground truth yield observations on which models can be trained and validated^6,7^. The inclusion of anonymised precision yield data within the CYCLeSS dataset addresses this gap, providing field-level observations of yield that can be used to assess the accuracy of models through comparison to the yield predicted by machine learning models. Secondly, development of a UK wide crop model has thus far been limited by the differing scale and coverage of available data of factors potentially impacting crop yield such as climate and soil conditions^5,26^. The CYCLeSS dataset addresses this data gap by aggregating and aligning climate, soil, and satellite data to the same 1 km^2^ grid. Since this climate, soil and satellite data is available for the entirety of the UK, the process of aggregation and alignment outlined in this study can be used by future researchers to efficiently retrieve data on grids beyond those included in the dataset in order to test model predictions on novel data. This process can also be used to guide retrieval of climate, soil and satellite data for areas where further yield data becomes available, allowing expansion of the model training and validation dataset, or fine-tuning of trained models. The dataset also provides information on temperature, rainfall, and soil type, which have been found to be among the most commonly used inputs into previous predictive machine-learning and deep-learning models for crop yield^5^.

This dataset can also be used to assess the validity of models built and parameterised using spatially more limited, but otherwise richer sources of data. The scope of the models that could be assessed in this way is broad, from (semi-)mechanistic, process-based models (such as APSIM^27^ or Sirius^28^) to more empirical, statistical regression models. Assessing how well such models can predict yield responses across a broader landscape is an important component of the development and out-scaling of these models to provide Digital Twins for crop productivity beyond the field or farm scales at which the models are usually developed^29^. For all models, the main limitations in the use of these datasets are the matching of the input variables for the model to the variables that are available for each of the locations in the dataset, and the availability of suitable values for any parameters that need to be set for each location^30^. The CYCLeSS dataset addresses this limitation by providing a link between precision crop yields, and the soil type and daily weather data associated with each location. Comparing model predictions to the observed yield data provided in the CYCLeSS dataset will then both allow the assessment of how widely a model can be implemented, and potentially identify the location-specific information that is required for the models to provide more reliable predictions. As an illustrative example, Addy *et al*.^31^ constructed a model for the impact of inter-annual weather variation on the yield response of winter wheat to applied nitrogen inputs using multiple years of yield data from the Broadbalk long-term experiment at Rothamsted Research. Modelling the impact of weather, aggregated within months for each growing season, allows a space-for-time substitution, and hence the prediction of winter wheat crop yields at different locations based on the observed weather in the growing season for each location^31^. As applied nitrogen inputs are not available for the precision crop yields, model predictions are based on optimal applied nitrogen levels^31^. Prediction errors can then be used to identify the need for model adjustments (re-parameterisations) for differences in crop characteristics at different locations, for example soil type, variety, or sowing date. Further models are being developed using data from the Broadbalk experiment to assess the impact of climate and nitrogen inputs on first (following a non-wheat crop) or second (following a first wheat crop) winter wheats rather than the continuous wheat crops considered in Addy *et al*.^31^.

There is also scope to improve existing crop models by assimilating satellite data into existing process-based models. This is because satellite data can provide measurements of crop parameters that many process-based models simulate via mathematical and statistical means. This concept is by no means new, and dates back to the availability of earth observation (EO)-derived measures of crop growth such as canopy leaf area index (LAI), some 40 years ago^32,33^. However, in recent years, increases in the spatial and temporal frequency of EO data capture have made these approaches increasingly viable, to the point where hybrid process-based and EO models can now be conceived for real time crop growth monitoring at the farm scale^34^. The assimilation of EO-derived metrics into process-based models confers several advantages^35^. Firstly, and most obviously, it corrects modelled data to observed data, by adjusting the time series of a given crop growth parameter (or multiple parameters) simulated by the model to match that measured by EO. This can be done at various points within a model, so that there are multiple ties back to observed data, increasing model accuracy. This is particularly valuable for accurate forecasting of yields within a given year, such as in systems designed to support real-time farm decision making. Secondly, EO data incorporates the impacts on crop parameters of factors such as local management history, fertiliser input, pests and diseases, unexpected weather events etc, all of which are challenging for process-based models to simulate and parametrise beyond a single site. Assimilation thus adjusts models from providing ‘potential’ yields excluding these factors closer to those likely to be observed in the field. The relationship between pre- and post-assimilation modelled yields can thus be used to adjust, and improve the accuracy, of model outputs even for spatial or temporal extents lacking EO data (e.g. forecasting future crop yields), as detailed by Hayman *et al*.^35^. Finally, assimilating EO data can allow models constructed and parametrised at the site level to be run effectively over larger spatial extents, by supplying parameters that would otherwise require assumptions. There are many approaches to EO data assimilation in crop yield models^35–38^ and the field is a rapidly developing one. CYCLeSS offers a potential route to explore and validate such approaches.

## Methods

### Gathering climate and soil data associated with crop yield

Metadata on climate conditions potentially associated with crop yield in the years 2015 to 2017 was taken from the ‘Climate, Hydrology and Ecology research Support System (CHESS) meteorology dataset for Great Britain’(10.5285/8baf805d-39ce-4dac-b224-c926ada353b7; 10.5285/b745e7b1-626c-4ccc-ac27-56582e77b900)^39,40^. This data covers the entirety of Great Britain at a temporal resolution of 1 day and a spatial resolution of 1 km^2^. The variables for which data was extracted from this dataset for inclusion in the CYCleSS dataset and their respective units of measurement are listed in Table 1.

**Table 1:** Variables in the ‘Climate, Hydrology and Ecology Research Support System (CHESS)’ dataset for which data was included in the CYCleSS dataset.

Variable	Unit
Near surface air temperature (1.2 m)	degrees K
Daily temperature range	degrees K
Precipitation – GEAR	kg m^−2^ s^−1^
Near-Surface Wind Speed (10 m)	m s^−1^
Surface Downwelling Shortwave Radiation	W m^−2^
Surface Downwelling Longwave Radiation	W m^−2^
Near-Surface Specific Humidity (1.2 m)	kg kg^−1^
Surface Air Pressure	Pa
Potential evapotranspiration over well-watered grass	mm/day
Potential evapotranspiration with interception correction	mm/day

Metadata on soil conditions potentially associated with crop yield for the years 2015 to 2017 was taken from the ‘Maps of indicators of soil hydraulic properties for Europe’ and ‘Mapping topsoil physical properties at European scale using the LUCAS database’ datasets (http://data.europa.eu/89h/jrc-esdac-39)^41,42^. These measurements were static for the duration of 2015 to 2017, with the ‘Maps of indicators of soil hydraulic properties for Europe’ dataset covering the entirety of the United Kingdom at a spatial resolution of 1 km^2^, and the ‘Mapping topsoil physical properties at European scale using the LUCAS database’ dataset covering the entirety of the United Kingdom at a spatial scale of 500 metres squared.

The variables for which data was extracted from the ‘Maps of indicators of soil hydraulic properties for Europe’ dataset for inclusion in the CYCleSS dataset and their respective units of measurement are listed in Table 2. Data on the following variables was extracted from the ‘Mapping topsoil physical properties at European scale using the LUCAS database’ dataset for inclusion in the CYCleSS dataset: Clay content (%), Silt content (%), Sand content (%), and coarse fragments (%) in topsoil (0–20 cm) were all modelled by Multivariate Additive Regression Splines. Bulk density was derived from soil texture datasets (obtained from the packing density, and the mapped clay content was modelled following the equation of Jones *et al*. 2003). USDA soil textural classes were derived from clay, silt and sand maps, and Available Water Capacity (AWC) for the topsoil fine earth fraction.

**Table 2:** Variables in the ‘Maps of indicators of soil hydraulic properties for Europe’ dataset for which data was included in the CYCleSS dataset.

Variable	Unit
Saturated water content	cm^3^/cm^3^
Water content at field capacity	cm^3^/cm^3^
Water content at wilting point	cm^3^/cm^3^
Saturated hydraulic conductivity	cm/day

### Aligning climate and soil data

Alignment of the gathered climate and soil metadata to the same spatial scale of 1 km^2^ grid squares was performed using R, and can be replicated using the file ‘**001_climate_and_soil_data_alignment.R**’ included in the code repository^43^. The CHESS meteorology dataset^39,40^ data for each variable was first loaded in its original format with separate files for monthly observations. These files were converted to the dataframe format and merged to create one large dataframe with all observations of each variable per year. The coordinates for locations of observations were then reprojected from the British National Grid to the WGS84 terrestrial reference system used in the ‘Maps of indicators of soil hydraulic properties for Europe’ dataset and ‘Mapping topsoil physical properties at European scale using the LUCAS database’ dataset using the ‘spTransform()’ method provided by the ‘rgdal’ package^44^. All reprojected coordinates were then rounded to seven significant figures to ensure consistency between the climate and soil datasets. Data from the CHESS meteorology dataset and data from both soil datasets were then aligned to the 1 km^2^ grid used in the ‘Maps of indicators of soil hydraulic properties for Europe’ dataset by merging the dataframes based on the ‘x’ and ‘y’ coordinates of each observation using the ‘merge’ function of the ‘dplyr’ package^45^. Finally, missing data were removed using the base R ‘na.omit’ function^43^. This resulted in a dataset containing observation of all climate and soil variables covering the entirety of Great Britain on a 1 km^2^ grid.

### Filtering for areas with arable land

The 1 km^2^ grid covering the entirety of Great Britain that was created when merging soil and climate data as described in the previous section on ‘Aligning Climate and Soil Data’ was filtered to include only grid squares containing arable land. This was achieved by overlaying the 1 km^2^ grid used in the soil and climate dataset described in the previous section on ‘Aligning Climate and Soil Data’ with the EUCROPMAP 2018 (http://data.europa.eu/89h/15f86c84-eae1-4723-8e00-c1b35c8f56b9)^46^ in QGIS^47^. The EUCROPMAP 2018^46^ assigns one of 21 land cover classes to each 10-by-10 metre area corresponding to the 10-by-10 metre pixel resolution of Sentinel-1 data. These land cover classes include 18 classes of arable crops; ‘common wheat’, ‘durum wheat’, ‘barley’, ‘rye’, ‘oats’, ‘maize’, ‘rice’, ‘triticale’, ‘other cereals’, ‘potatoes’, ‘sugar beet’, ‘other root crops’, ‘sunflower’, ‘rape and turnip rape’, ‘soya’, ‘dry pulses’, ‘fodder crops’, and ‘bare arable land’. The QGIS raster calculator was used to determine the grid squares containing one or more of the aforementioned arable land cover classes, excluding bare arable land. After filtering there remained 125016 grid squares containing arable land for which CHESS meteorology data, ‘Maps of indicators of soil hydraulic properties for Europe’ data, and ‘Mapping topsoil physical properties at European scale using the LUCAS database’ data was available for 2015–2017. These datapoints were primarily concentrated in the south of England, but also included coastal areas of Wales, Northern England, and Scotland. The percentage of arable land within each grid square was included in this dataset as well as the soil hydraulic properties derived from the ‘Maps of indicators of soil hydraulic properties for Europe’ dataset, top soil physical properties derived from the ‘Mapping topsoil physical properties at European scale using the LUCAS database’ dataset, and daily observations of climate data derived from the CHESS meteorology dataset. The detailed steps for conducting this process in QGIS are included in the figshare repository^48^ (**Large Scale Modelling Training Dataset Methods.docx**).

### Filtering for areas with available crop yield data

**Fig. 1:**
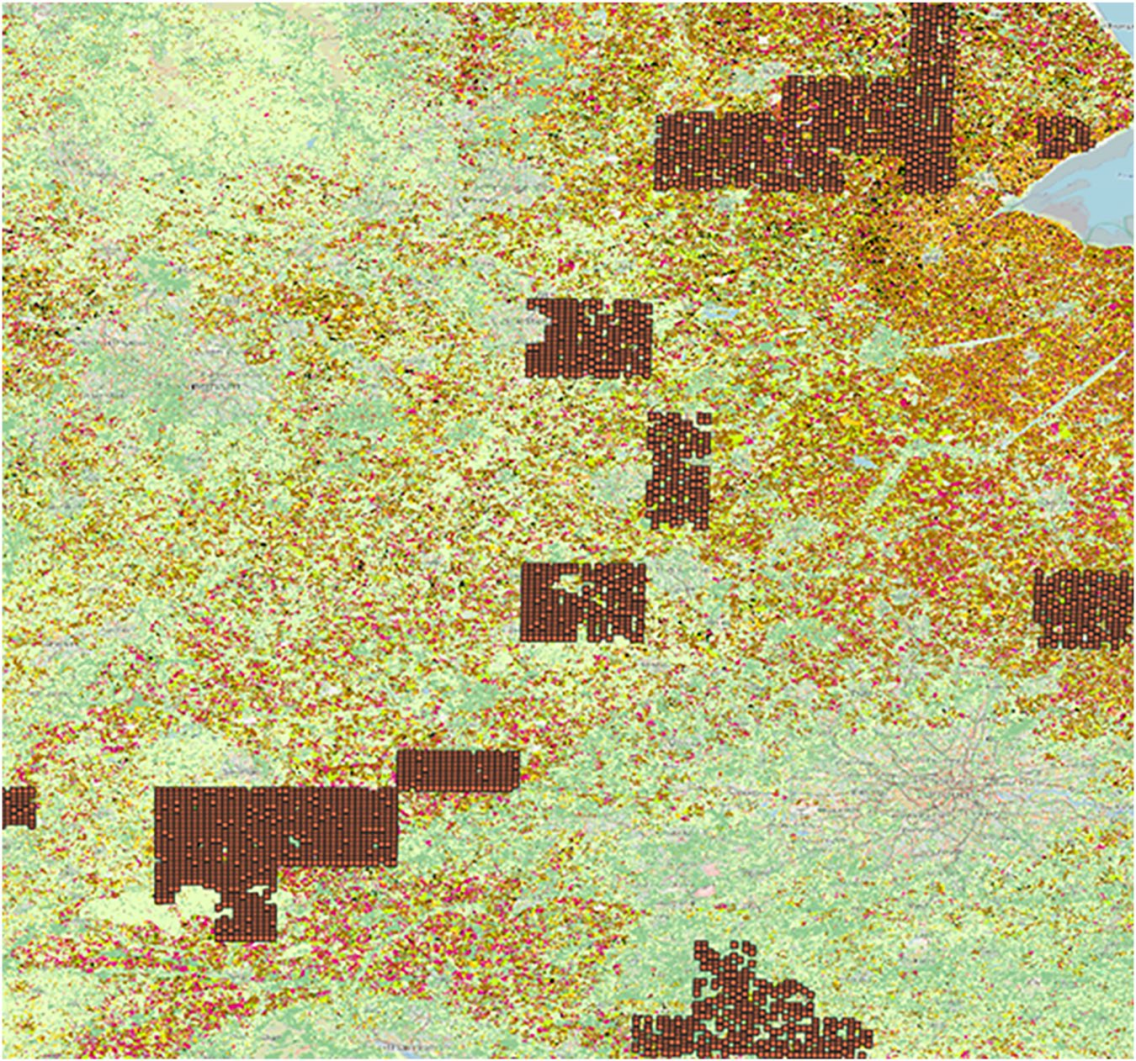
The 1 km^2^ grid squares with available data climate and soil conditions as well as precision yield data for years 2015–2017 overlayed on EUCROPMAP 2018 land cover class data.

The data were further filtered to obtain a dataset consisting of only grid squares containing arable land for which UK Centre for Ecology and Hydrology Achieving Sustainable Agricultural Systems (ASSIST) Precision Yield Dataset^21^ was available in addition to climate data from the CHESS meteorology dataset and soil data from the ‘Maps of indicators of soil hydraulic properties for Europe’ dataset, and ‘Mapping topsoil physical properties at European scale using the LUCAS database’ dataset. This was achieved by aligning the 1 km^2^ grid squares derived as described in the previous section on ‘Filtering for areas with arable land’ with a raster of 10 km^2^ square areas known to contain fields for which UKCEH ASSIST Yield Data for the years 2015–2017 were available in QGIS. This 10 km^2^ buffer zone ensured the exact locations of fields from which UKCEH ASSIST Yield Data was collected remained sufficiently anonymous, as the average UK farm size is 88 hectares, meaning a 10 km square will on average contain over 100 farms such that the risk of a user being able to associate yield data with any one contributing farm is minimal^49^. This resulted in a dataset containing 3070 1 km^2^ grid squares as seen in Fig. 1. A polygonal mask corresponding to the extent of the area of these 3070 1 km^2^ grid squares was then created and used to guide extraction of Sentinel 1 Satellite time series data as described in the following section: ‘Extracting satellite data’. Detailed instructions describing how to carry out the filtering and mask creation described in this section are included in the figshare repository^48^ (**Large Scale Modelling Training Dataset Methods.docx**).

**Fig. 2:**
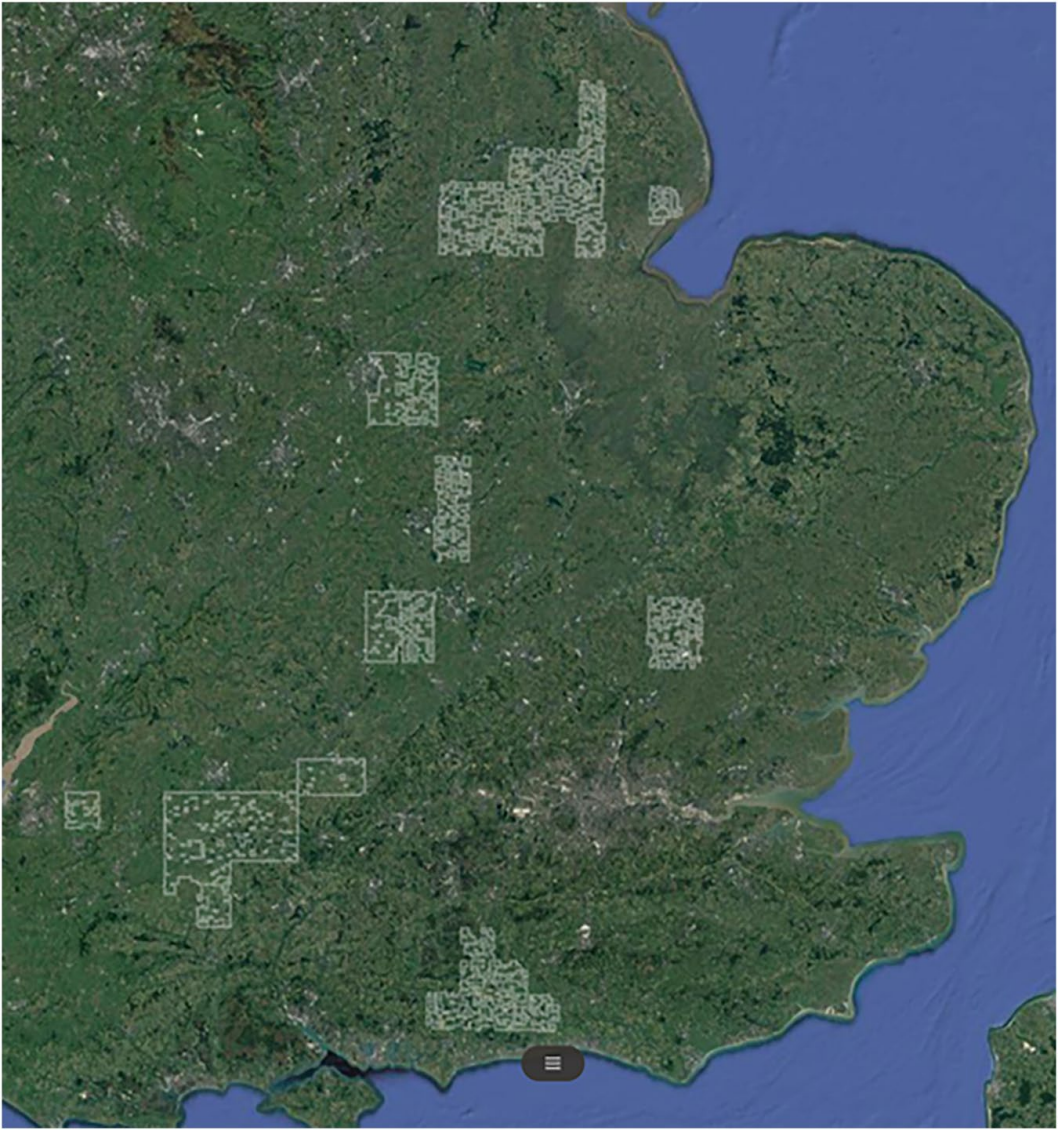
Extent of 1 km^2^ grid squares with available data on climate, soil and precision yield data for which time-series of Sentinel-1 synthetic aperture radar data for 2015–2017 was extracted using the SEPAL cloud computing platform.

### Extracting satellite data

Time-series of Sentinel-1 Synthetic Aperture Radar (SAR) data band values were then extracted for the extent of the 3070 grid squares for containing arable for which climate and soil data was available for the years 2015–2017, and which fell within the 10 km^2^ areas surrounding fields from which ASSIST yield and crop type data was collected in 2015–2017. Satellite data was extracted using the SEPAL cloud computing platform which enabled upload of a polygonal mask marking the boundaries of the grid extent so that data could be extracted for only 10-by-10m pixels within these areas^50^. The Sentinel-1 SAR data extracted included 5-daily time-series of radar band values collected simultaneously at two different polarisations: ‘VV’ and VH’, as well as the ratio between these values at each time step. ‘VV’ band values represent radar signals that were both emitted and received in vertical polarisation, a type of scattering that is often associated with bare soil or open water^51^. ‘VH’ band values represent radar signals emitted in vertical polarisation and received in horizontal polarisation, a type of scattering often caused by leaves, branches, or forest canopy^52^. These dual-polarity radar band values and the ratio between them have been used in several studies to successfully classify crop type^53–56^ and to monitor crop growth as dual-polarity VV and VH backscatter has been shown to be very sensitive to variations in crop height and biomass and is not affected by cloud cover^51,52,57,58^. Sentinel-1 SAR radar band time-series data was extracted via the SEPAL platform in three month chunks, which were then converted to yearly dataframes using the ‘merge’ function of the ‘dplyr’ package in R^45^. Code for performing merging of 3-monthly chunked Sentinel-1 SAR radar data is provided in the ‘**002_merge_sentinel1.R**’ file in the code repository.

### Merging climate, soil, and satellite data with data on crop yield and crop type

Precision yield data remain the property of the individual land managers and are used in this manuscript under non-disclosure agreements permitting anonymised use for research purposes. The data were anonymized by UKCEH for this publication, in accordance with these agreements, to remove data holder information and aggregate spatial locations.

To ensure the exact location of fields for which ASSIST yield and crop type data was collected remained anonymous, data was passed between and handled by two isolated teams for certain steps in the process of merging the dataset of 3070 1 km^2^ grid squares with aligned Sentinel-1 SAR radar data, CHESS meteorology dataset climate data, and ESDAC soil data with data on crop type and yield. This included one team of collaborators based at The Alan Turing Institute, Rothamsted Research, John Innes Centre and University of Exeter (‘Food Security Project Team’) and a team of collaborators based at UK Centre for Ecology and Hydrology (‘UKCEH Team’).

The Food Security Project Team provided the UKCEH Team with the Sentinel-1 SAR radar band data extracted as described in section 2.5 and associated on climate and soil conditions for all 10-by-10 metre satellite pixels within the 3070 1 km^2^ grid squares that overlapped with the 10 km square areas with ASSIST yield data for 2015 to 2017. This data was provided to the UKCEH Team in a standard data frame (.csv) format. The location of each 10-by-10 metre satellite pixel in the dataset was provided to the UKCEH Team in the form of latitude and longitude on the WGS84 coordinate system.

The UKCEH Team found matches between the locations of the satellite pixels and locations of fields for which ASSIST yield data is available for 2015 to 2017, filtering the data to include only pixels with fields where yield data is available. The UKCEH Team then aggregated data from the 10-by-10 metre pixels to field level, calculated the average satellite band data values and provided crop yield in mean tonnes per hectare per field for each crop type for 2015 to 2017. The UKCEH Team then removed all data on field location at 1 km^2^, provided 10 km^2^ British National Grid reference coordinates for each field and returned a final dataset containing mean 5-daily Sentinel-1 SAR radar band values, mean yield per field for each crop type, associated data on climate and soil conditions, and a random ID number for each field. As the climate and soil was previously aggregated to 1 km^2^ scale grid of 3070 grid squares by the Food Security Project Team, the location of fields in this final dataset were not identifiable at any finer resolution than this 1 km^2^ grid, even via reverse engineering by identifying locations of soil and climate combinations.

The final merging process outlined above, including anonymisation of field locations, can be replicated on dummy data using the contents of **‘CYCLESS_anonymisation.zip’** included in the dataset code GitHub repository.

## Data Overview

The finalised dataset contains data from 934 areas of arable land in England, each measuring 1 km^2^ in size, where crop plants were grown between 2015–2017, hereafter referred to as ‘grid squares’. Each grid square is given an individual identification number that links the data on the grid square location, the crop type grown within it each year, the yearly mean crop yield per field, time series of Sentinel-1 radar band data collected at 5-day intervals averaged over the 1 km^2^ area, as well as data on climate and soil conditions for each grid square. This dataset with ground-truth precision yield data is therefore suitable for training and validating a machine learning model of crop yield prediction. We would like to emphasise that the satellite data, climate data, and soil data are all publicly available for any area in the UK. Data is available for 201 grid squares in 2015, 292 grid squares in 2016, and 443 grid squares in 2017 (Table 3). The dataset primarily contains grid squares where winter wheat was grown, followed by grid squares where oilseed rape and spring barley was grown, and a smaller number of grid squares where winter barley and beans were grown (Table 3).Data on yield for crops present within less than 10 grid squares has been omitted from the final dataset to ensure the location of farms on which these rarer crops are grown remains anonymous.

**Table 3:** Number of 1 km grid squares in dataset with available climate, soil, crop yield and Sentinel-1 radar band data by year and crop type.

Crop Type	Number of 1 km^2^ grid squares (2015)	Number of 1 km^2^ grid squares (2016)	Number of 1 km^2^ grid squares (2017)
Winter Wheat	98	158	216
Winter Barley	17	24	44
Spring Barley	37	40	29
Oilseed Rape	37	60	119
Beans	12	10	35
Total	201	292	443

## Data Records

Data comprising the final CYCleSS dataset is available through Figshare repository (10.6084/m9.figshare.27225807)^48^. The main folder provided (**‘CYCLeSS_dataset_141024’**) is divided into three subfolders: the **‘crop_yield_type_and_satellite_data’** subfolder contains data on crop yield per field, crop type, and Sentinel-1 radar band data for grid squares, the **‘climate_data’** subfolder contains all climate data extracted and aggregated to 1 km^2^ scale from the CHESS meteorological dataset for each grid square, and the **‘soil_data’** subfolder contains data on soil conditions extracted from the ‘Maps of indicators of soil hydraulic properties for Europe’ dataset, and ‘Mapping topsoil physical properties at European scale using the LUCAS database’ dataset for each grid square. For each grid square data is linked across all subfolders and files by a unique identification number found in the ‘ID’ column of each file.

The subfolder ‘**crop_yield_type_and_satellite_data**’ contains data on crop type, mean yield per field calculated in tonnes per hectare for each field, and 5-daily time series of Sentinel-1 radar band data for each grid square. The.csv files within this folder contain this data split by radar band (‘VV’, ‘VH’ and the ratio between these values, referred to simply as ‘Ratio’) and year, designated by the following file name format; ‘**Band_Year_MeanYieldperField.csv**’. For example, the file ‘**VV_2015_MeanYieldperField.csv**’ contains data on crop type, mean yield per field, and 5-daily ‘VV’ radar band data for the year 2015. Within each.csv file the **‘east’** and **‘north’** columns provide the six-digit easting northing coordinates for the 10 km^2^ British National Grid square containing each field, with the **‘grid_ID’** column providing a unique identification number for each of these grid squares. The **‘ID’** column denotes the individual field identification number, the **‘Year’** column denotes the year data was collected, the **‘Crop’** column contains the crop type grown within each grid square, and the **‘Yield’** column contains the mean yield per field of each crop type for each grid square. Time-series of the relevant Sentinel-1 radar band value denoted by the file name is then included in a series of columns with names in the following format; **‘XYear.Day.Month’** where for instance a column with the name **‘X2015.01.03’** would contain the band value on 1st March 2015.

Data on climate conditions for each grid square is provided in the **‘climate_data’** subfolder, with data further divided into sub-folders by year from 2015 to 2017. Data is then divided into individual.csv files containing time-series of a single variable extracted from the CHESS meteorological dataset for each year with the name format **‘variable_year.csv’**. For instance, the file **‘dtr_2015.csv’** contains daily time series data on the daily temperature range for each 1 km^2^ grid square. See Table 4 for the complete list of sub-folder names and the corresponding variable the.csv files within contain data on. For each.csv the unique identification number of each field is given in the **‘ID’** column, and a unique identifier for the 10 km^2^ British National grid square each field was contained within is provided in the **‘grid_ID’** column, along with columns containing the six-digit easting (**‘east’**) and northing (**‘north’**) of that grid square. The variable measurement for each day is given in columns with the format **‘XYear.Day.Month’** where for instance a column with the name **‘X2015.01.03’** would contain the observed value for the variable on 1st March 2015.

**Table 4:** Abbreviation for data on each variable extracted from the ‘Climate, Hydrology and Ecology research Support System (CHESS)’ dataset used as file names.

Variable	Abbreviation/File name
Near surface air temperature (1.2 m)	‘tas’
Daily temperature range	‘dtr’
Precipitation – GEAR	‘precip’
Near-Surface Wind Speed (10 m)	‘sfcWind’
Surface Downwelling Shortwave Radiation	‘rsds’
Surface Downwelling Longwave Radiation	‘rlds’
Near-Surface Specific Humidity (1.2 m)	‘huss’
Surface Air Pressure	‘psurf’
Potential evapotranspiration over well-watered grass	‘pet’
Potential evapotranspiration with interception correction	‘peti’

Data on soil conditions for each grid square is provided in the **‘soil_data’** subfolder. This data is divided into two.csvs by year, with **‘LandUseandSoil_2015_2016’** containing data extracted for each grid square for both 2015 and 2016, and **‘LandUseandSoil_2017’** providing data extracted for 2017. Within each.csv file the **‘ID’** column provides a unique identification number for each field, **‘grid_ID’** provides a unique identification number for the 10 km^2^ square area of the British National Grid containing the field, the **‘east’** and **‘north’** columns provide the easting northing coordinates of each grid square. The **‘p_arable’** column provides the percentage of arable land within each grid square derived from the EUCROPMAP 2018 land cover class dataset. The rest of the columns provide data on variables extracted from the ‘Maps of indicators of soil hydraulic properties for Europe’ dataset, and ‘Mapping topsoil physical properties at European scale using the LUCAS database’ dataset. See Table 5 for a key to abbreviations used as column names for each variable^59^.

**Table 5:** Abbreviation for data on each variable extracted from the ‘Maps of indicators of soil hydraulic properties for Europe’ dataset, and ‘Mapping topsoil physical properties at European scale using the LUCAS database’ dataset used as column names.

Variable	Abbreviation/Column name
Available Water Capacity for the topsoil fine earth fraction	‘awc’
Bulk density derived from soil texture datasets	‘bd’
Coarse fragments (%) content in topsoil modelled by Multivariate Additive Regression Splines,	‘cf’
Clay content (%) in topsoil (0–20 cm) modelled by Multivariate Additive Regression Splines	‘clay’
Water content at field capacity	‘fc’
Saturated hydraulic conductivity	‘ks’
Sand content (%) in topsoil modelled by Multivariate Additive Regression Splines,	‘sand’
Silt content (%) in topsoil modelled by Multivariate Additive Regression Splines	‘silt’
USDA soil textural classes derived from clay, silt and sand maps	‘text’
Saturated water content	‘ths’
Water content at wilting point	‘wp’

## Technical Validation

### Comparison of soil and climate conditions for CYCLeSS dataset to UK cropland

**Fig. 3:**
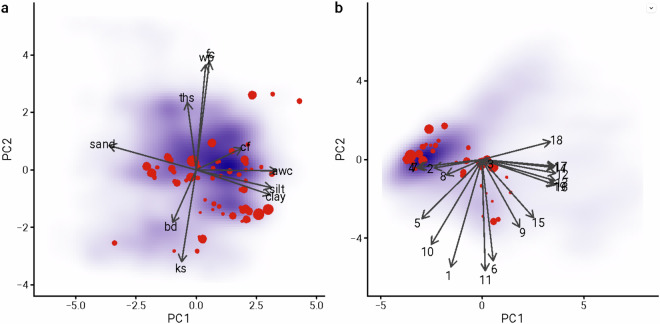
Principal components analysis of (**a**) soil variables and (**b**) BIOCLIM bioclimatic variables for CYCLeSS sites (red circles) and UK cropland (blue shading). Circle area is proportional to number of samples. Shading reflects frequency of soil and climate conditions across UK cropland.

We compared soil properties and historical climate (1991–2020 mean) for CYCLeSS sites against UK cropland as a whole using principal components analysis (Fig. 3). A random sample of 6000 (of a total of 125,016) cropland 1 km^2^ grid cells was used for comparison. The 19 BIOCLIM bioclimatic variables were calculated from HAD-UK historical monthly minimum and maximum temperature and precipitation using the *bioclim* package for R^60,61^. We found that CYCLeSS sites gave a good representation of general variation in both soil and climate across UK cropland, though with some bias toward denser, more clayey soils and more seasonal (BIO4, BIO7) sites with a greater maximum temperature (BIO5).

### Cleaning and standardisation of precision yield data

Prior to inclusion in the CYCLeSS dataset, data points were excluded from the precision yield data if they were collected under conditions outside the reliable working parameters of the combine harvesters, originated from outside known cropping areas, or fell outside the range of biologically possible yields^21^. The precision yield data was further refined by removing potentially inaccurate data points that were more than two standard deviations away from either the field mean or the local mean, as determined by the 10 closest data points^21,61^. To account for calibration differences between two combine harvesters operating in the same field within the same year, the combined mean data from both machines was used^21^. Data was excluded when more than two combine harvesters were used to collect data from the same field^21^.

## Usage Notes

### Limitations of the data

Although integral to the potential usefulness of this dataset in developing and validation models of a crop yield, the inclusion of precision yield data within the CYCLeSS dataset introduces a number of factors that require consideration. Precision yield data can be highly variable in their format, method of measurement, precision and accuracy^62^. Different manufacturers use different yield measurement systems, and individual farmers vary in terms of the effort put into calibrating and checking the accuracy of their equipment^63^. The accuracy of the data can also be further compromised by how the equipment is used by the operator (e.g. harvesting at below the full swatch width, not turning off the yield monitor when not harvesting, inaccurate readings at high speeds or when turning)^63^. Although such variation is accounted for to some extent when cleaning the data to produce the dataset used in this study^20^, and is unlikely to result in a systemic bias, it does introduce a level of uncertainty that may overwhelm any useful signal, especially for crops with only a few sampled fields, such that models built on these data may have limited predictive ability.

A further limitation comes from the way in which precision yield data were sourced. Whilst fields were scattered across the arable areas of Southern England, they are unlikely to be representative of yields under all climatic and environmental contexts. Equally, farms with accessible precision yield data are likely to be those that have invested in modern equipment with onboard yield monitors, and to have the facility to access and make available these data – there may be types of farm practice and farmer attitudes associated with such farms that in turn affect transferability of the modelled results to other farm systems.

Currently the only Sentinel satellite data included are synthetic aperture radar data bands, while other Sentinel data bands such as RGB imagery and NDVI may be beneficial in identifying the growth of agriculture crops^64,65^. The process of retrieving and merging Sentinel satellite data with data on climate, soil conditions and precision yield data outlined in the methodology of this study could be highly beneficial in guiding the retrieval of these other relevant bands to further explore their usefulness as inputs into models of crop yield.

## Data Availability

All data comprising the final CYCleSS dataset is available through Figshare repository (10.6084/m9.figshare.27225807)^48^. See the Data Records section for a detailed breakdown of the contents of this repository. Researchers who are further interested in the underlying data should contact the authors affiliated with UKCEH.

## Code availability

R code used to merge and align available UK climate, soil, and Sentinel-1 synthetic aperture radar data to the same 1 km^2^ grid is provided in the following GitHub repository: https://github.com/alan-turing-institute/CYCLeSS-dataset-code. Dummy data and code needed to replicate the final process of merging climate, soil, and satellite data with UKCEH precision yield data and anonymisation of field locations is contained within the ‘CLYCESS_anonymisation.zip’ folder shared as part of this repository. R version 4.2.3 was used for the creation of this dataset.
